# Identification of Key Volatile Compounds in Tilapia during Air Frying Process by Quantitative Gas Chromatography–Ion Mobility Spectrometry

**DOI:** 10.3390/molecules29184516

**Published:** 2024-09-23

**Authors:** Tianyu Chen, Yong Xue, Chunsheng Li, Yongqiang Zhao, Hui Huang, Yang Feng, Huan Xiang, Shengjun Chen

**Affiliations:** 1 College of Food Science and Engineering, Ocean University of China, Qingdao 266003, China; chentianyu5220@stu.ouc.edu.cn (T.C.); xueyong@ouc.edu.cn (Y.X.); 2 Key Lab of Aquatic Product Processing, Ministry of Agriculture and Rural Affairs of the People’s Republic of China, National Research and Development Center for Aquatic Product Processing, South China Sea Fisheries Research Institute, Chinese Academy of Fishery Sciences, Guangzhou 510300, China; zhaoyq@scsfri.ac.cn (Y.Z.); huanghuigd@aliyun.com (H.H.); fengyang@scsfri.ac.cn (Y.F.); xianghuan@scsfri.ac.cn (H.X.)

**Keywords:** tilapia, air frying, oxidation of proteins and lipids, volatile compounds, quantitative GC-IMS, correlation network

## Abstract

Air frying as a new roasting technology has potential for roasted fish production. In this study, the changes in volatile compounds (VCs) during air frying of tilapia were studied by quantitative gas chromatography–ion mobility spectrometry, followed by the identification of key VCs based on their odor activity value (OAV). There were 34 verified VCs, of which 16 VCs were identified as the key VCs with OAV ≥ 1. Most of the VCs were improved by air frying and peaked at 20 min. During the air frying, the total sulfhydryl content markedly decreased, while the protein carbonyl and MDA content significantly increased, suggesting the enhancement in the oxidation of lipids and proteins. The correlation network among the chemical properties and key VCs was constructed. The change in total sulfhydryl, protein carbonyl, and MDA showed significant correlation with most of the key VCs, especially 2-methyl butanal, ethyl acetate, and propanal. The results indicated that the oxidation of lipids and proteins contributed the most to the flavor improvement in air-fried tilapia. This study provides a crucial reference for the volatile flavor improvement and pre-cooked product development of roasted tilapia.

## 1. Introduction

As the third largest freshwater aquaculture fish, tilapia has the advantages of fast growth and large yield around the world due to the strong environmental adaptability [1,2]. It is rich in nutrients such as unsaturated fatty acids and proteins, and is usually processed into frozen filets because of the lack of intermuscular spines [3]. However, this primary processing method leads to low added value of tilapia. Therefore, there is a great need for an alternative technique, which can obviously improve the added value of tilapia.

Roasting as a high-value processing method of aquatic products can significantly improve the flavor and added value of fish. Traditional roasting methods of fish such as oven-roasting, barbecuing, and deep-frying often need a lot of oil and easily produce harmful substances such as polycyclic aromatic hydrocarbon, acrylamide, and heterocyclic amine [4,5,6], unfitting of the needs of a modern healthy diet. Air frying as a new roasting technology possesses high heat transfer efficiency because of the hot air circulating in the chamber [7]. During air frying, the high-temperature airflow surrounds the food, which simultaneously induces a fast mass transfer in a transient state among the hot air medium, water, and oil inside the food [8]. Therefore, it can effectively reduce the oil content and produce a similar product appearance with deep-fat frying [9,10], leading to the good texture and mouthfeel of products. Recently, the air frying technology has been applied among plant products [11], chicken [12], and surimi products [13,14], but there is a lack of application in fish, especially tilapia.

Aroma is one of the main quality characteristics of roasted fish, resulting from the existence of volatile compounds (VCs) as essential components in fish. VCs are usually caused by chemical reactions such as the oxidative decomposition of lipids and proteins in fish during roasting, and their species and contents change significantly along with the roasting [15]. However, few studies have focused on the change in VCs during the air frying of tilapia. As a new detection technology for VCs, gas chromatography–ion mobility spectrometry (GC-IMS) possesses more sensitive, accurate, and faster detection capabilities than gas chromatography–mass spectrometry (GC-MS) due to the fast response ability of IMS [16]. However, the GC-IMS is usually used for the qualitative analysis of VCs to compare among different groups, which is hardly used to determine the key VCs in food.

Therefore, in this study, the VCs of tilapia were identified and quantified by quantitative GC-IMS, followed by the selection of key VCs based on the calculation of the odor activity value (OAV). The contents of malondialdehyde (MDA), total sulfhydryl, and protein carbonyl were also analyzed. The relationship among the key VCs and oxidative indexes of proteins and lipids was studied by a correlation analysis to determine the possible formation mechanism. This study is expected to provide a novel method to enhance the added value of tilapia, and offer comprehensive information for the flavor change in air-fried tilapia in industrial production.

## 2. Results and Discussion

### 2.1. Change in VCs during Air Frying by Quantitative GC-IMS

**Figure 1:**
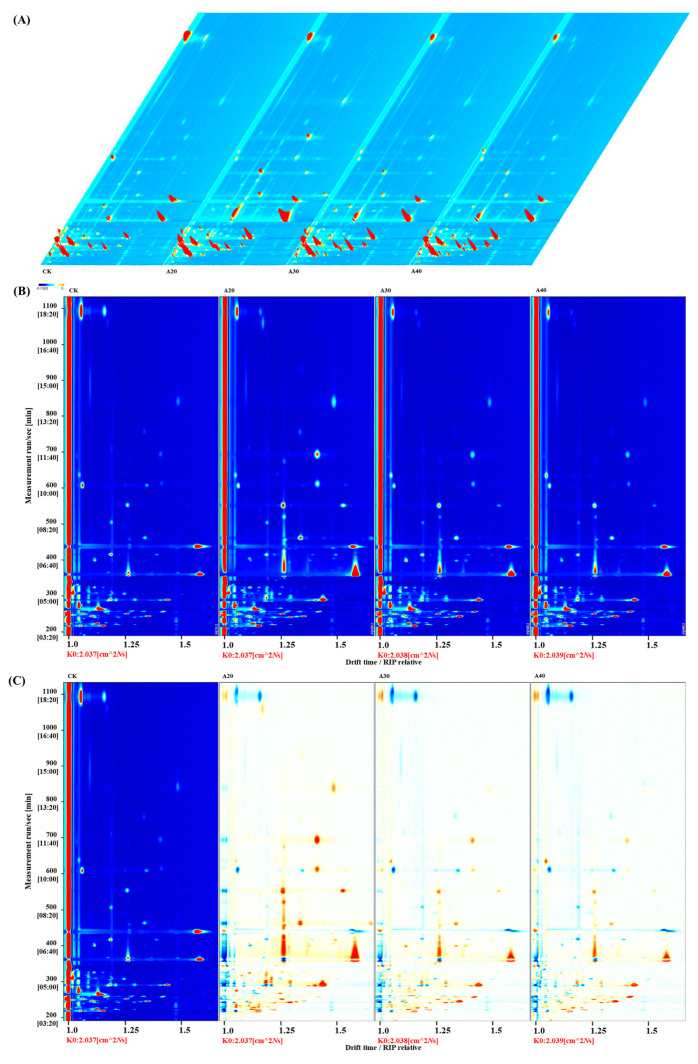
Identification of VCs by GC-IMS; (**A**) 3D topographic plot, (**B**) topographic plot, and (**C**) topographic subtraction plot.

Quantitative GC-IMS was used to analyze the variation in VCs during air frying of tilapia. The three-dimensional plots of VCs in the CK, A20, A30, and A40 groups are shown in Figure 1A. The intensities of peak signals among the A20, A30, and A40 groups were highly similar, and much higher than those in the CK group. To further display the difference in VCs in types and concentrations, the two-dimensional plots were displayed after the normalization of 3D topographical visualizations (Figure 1B). The signal for most VCs appeared in similar locations in all groups and concentrated on the period of 200–900 s. The topographic subtraction plot was obtained by subtracting the signals of the CK group from those of the other groups to compare the difference in VCs’ content among the four groups (Figure 1C). The blue and red represent that the concentration of VCs was less and higher than that in the raw tilapia, respectively. The results suggested that most VCs were improved by air frying, especially in the A20 and A30 groups.

**Figure 2:**
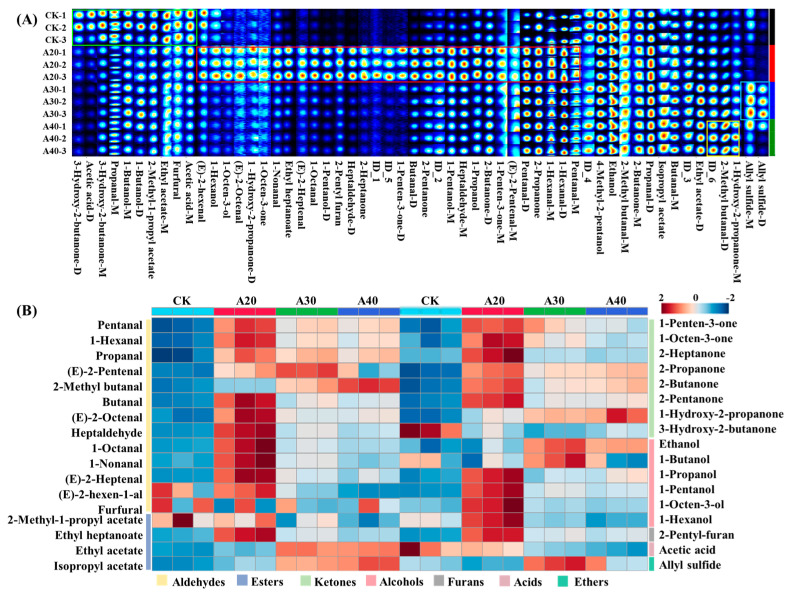
Change in VCs during air frying by quantitative GC-IMS. (**A**) Fingerprint of VCs and (**B**) concentration heatmap of VCs.

**Figure 3:**
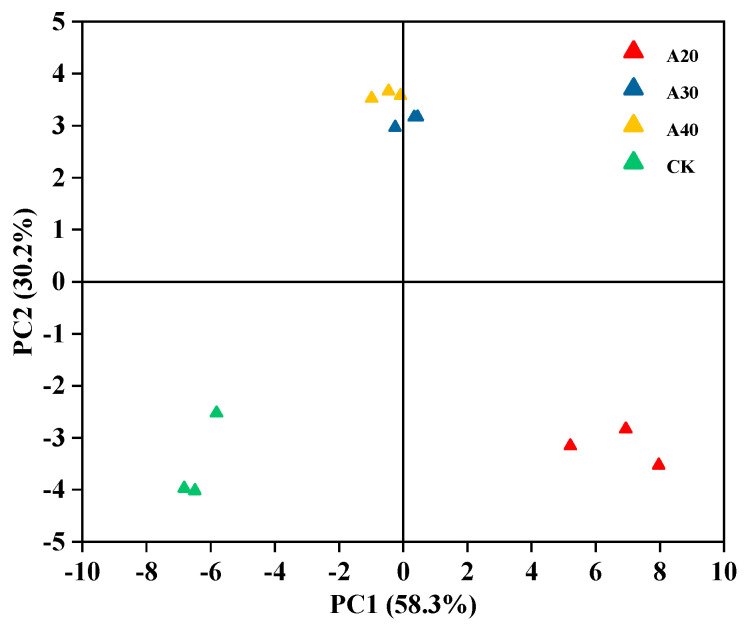
Similarity analysis of VCs during air frying of tilapia by PCA.

The VCs were further identified using the NIST database (Figure S1 and Table S1). There were 56 VCs (including 1 internal standard substance) in the process of air frying. A total of 15 VCs appeared as both a monomer and dimer, and 6 VCs were not determined. The fingerprint plot was drawn using all the peaks to compare the content of VCs in the tilapia during the process of air frying (Figure 2A). Most VCs were concentrated in the A20 group, and the abundance and species of VCs in the A30 and A40 groups were similar. The content of VCs was further calculated according to the content of the internal standard substance after the monomer and dimer combination (Table S2). There were 34 VCs during air frying of tilapia including 13 aldehydes, 8 ketones, 6 alcohols, 4 esters, 1 acid, 1 furan, and 1 ether. As shown in Figure 2B, the content of VCs in air-fried tilapia was significantly higher than that in raw tilapia, especially in the A20 group, where most VCs (22 kinds) reached the maximum. In addition, the concentration of (E)-2-pentenal, ethyl acetate, ethanol, 1-butanol, and allyl sulfide peaked in the A30 group, and the concentration of 2-methyl butanal, isopropyl acetate, and 1-hydroxy-2-propanone peaked at the end of air frying (A40). Generally, the VCs of aldehydes, alcohols, and ketones are first produced by the oxidative decomposition of proteins and lipids under high temperature, and the esters are then produced by an esterification reaction with acids and alcohols. In this study, high contents of aldehydes, alcohols, and ketones were found at the initial stage of air frying, while high contents of esters were observed at the later stage of air frying. Similar results were found in the oven-roasted fish [15]. The difference in VCs in different groups was further distinguished by PCA (Figure 3). The variance contribution rates of PC1 and PC2 were 58.3% and 30.2%, respectively, which were enough to reflect the whole difference in VCs in different groups. The VCs in the CK group or A20 group showed more difference with the other groups, while there was a strong similarity between A30 and A40 groups, suggesting that the air frying could apparently influence the contents of VCs in tilapia.

### 2.2. Identification of Key VCs during Air Frying

Aldehydes are generally considered as the main flavor source of aquatic products due to the low threshold, and most aldehydes have pleasant aroma such as floral, fruity, and malty odor [3,17]. In this study, aldehydes were the most abundant in the air frying process, and most aldehydes peaked in the A20 group (Table S2). The aldehydes with the most abundance included 1-hexanal, pentanal, and propanal, reaching the maximum contents of 2.791, 0.885, and 0.600 mg/kg in the A20 group, respectively. The OAV is usually used to screen the key VCs in aquatic products [18]. The VCs with OAV ≥ 1 are considered as the key VCs that vitally impact on the overall flavor of aquatic products [19]. In this work, there were nine aldehydes with an OAV ≥ 1 (Table 1). The highest OAV was found in 1-hexanal with grassy and fruity odor, reaching 620.18 in the A20 group. The highest OAV for 1-hexanal has also been found in the oven-roasted tilapia [15]. High OAV of 1-nonanal, 1-octanal, heptanal, and pentanal was also observed during air frying of tilapia, especially after air frying for 20–40 min, resulting in the formation of pleasant fatty, green, herbal, and fruity aroma in air-fried tilapia. These aldehydes were also found as the key VCs in the fermented tilapia surimi [3,18]. In addition, the OAVs of (E)-2-octenal, (E)-2-heptenal, and propanal were all improved after air frying, contributing to the increase in fruity and fatty flavor [20]. The 2-methyl butanal was significantly enhanced after air frying and reached the maximum of OAV over 200 at the end of air frying, leading to the increase in malty and almond aroma of tilapia. This aldehyde is also considered as a vital contributor for the flavor of oven-roasted tilapia [15].

Ketones generally play a coordinating role in the overall volatile flavor of meat products [21]. In this work, ketones were the second most diverse VCs after aldehydes, and most ketones reached the maximum in the A20 group. Among these ketones, 2-butanone and 2-propanone had the highest content, reaching 0.423 and 0.275 mg/kg in the A20 group, respectively. There were three ketones with OAV ≥ 1, including 1-octen-3-one, 3-hydroxy-2-butanone, and 1-penten-3-one (Table 1). Generally, 1-octen-3-one is found as a key VC in aquatic products because of its mushroom aroma and low threshold [3]. In this study, 1-octen-3-one had the highest OAV (>1700), playing an important role in the overall flavor of air-fried tilapia. The OAV of 1-penten-3-one first increased and then decreased during air frying, influencing the flavor of tilapia after air frying. Differently, the content of 3-hydroxy-2-butanone significantly decreased after air frying, probably resulting from the destruction of the alkoxy radical of 3-hydroxy-2-butanone at high temperature [21].

The content of most alcohols in the tilapia significantly increased after air frying, and the alcohols with the highest content were ethanol (0.688 mg/kg) and 1-pentanol (0.413 mg/kg) in the A30 and A20 groups, respectively. The high concentrations of ethanol and 1-pentanol have also been found in the oven-roasted tilapia [15]. According to an OAV ≥ 1, 1-octen-3-ol and 1-pentanol were identified as the key alcohols and all reached their maximum in the A20 group (Table 1). As an unsaturated alcohol, 1-octen-3-ol provides a mushroom flavor and also serves as a key VC in other aquatic products [22,23]. The OAV of 1-pentanol was less than 1 in raw tilapia but exceeded 1 after air frying, contributing to the increase in green and grassy flavor.

Esters are generally synthesized by esterification reactions between alcohols and acids or transesterification reactions [18,24], and make an important contribution to the aroma formation of many products such as yak meat [21], silver carp surimi [25], and Baijiu [26]. In this study, there were four esters in the process of air frying of tilapia. Ethyl acetate was the most abundant ester during air frying, and reached the maximum of 0.458 mg/kg in the A30 group. Ethyl heptanoate was another abundant ester and peaked in the A20 group (0.346 mg/kg). Moreover, the above two esters were also identified as the key VCs with OAV > 1 (Table 1). Ethyl acetate is considered as one of the most important esters in aquatic products [27,28,29]. In this study, the improved ethyl acetate and ethyl heptanoate by air frying contributed to the increase in fruity and floral flavor of tilapia.

### 2.3. Change in Chemical Properties of Tilapia during Air Frying

**Figure 4:**
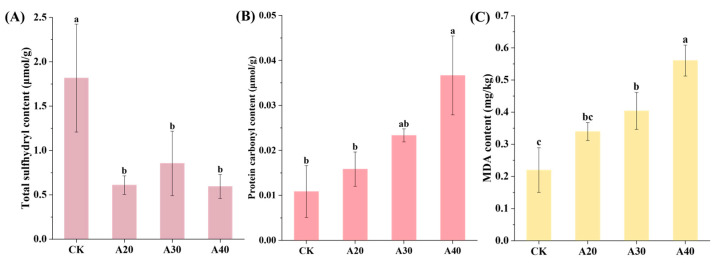
Change in chemical properties of tilapia during air frying. (**A**) Total sulfhydryl content, (**B**) protein carbonyl content, and (**C**) MDA content. Data are shown as mean ± standard deviation (n = 3). Different lowercase letters (a–c) within different groups indicate significant differences by one-way analysis of variance with Tukey test (*p* < 0.05).

Generally, most VCs are derived from the thermal degradation and oxidation of lipids and proteins [30]. Therefore, the change in the oxidation of lipids and proteins was studied during air frying of tilapia. The sulfhydryl groups in cysteine residues can be easily oxidized into disulfide bonds by free radicals, resulting in the decrease in sulfhydryl group content [31]. Thus, the total sulfhydryl content is usually regarded as a representative index to evaluate the degree of protein oxidation. In this work, the total sulfhydryl content of tilapia significantly decreased after air frying, indicating that the protein oxidation deepened with the increase in air frying time (Figure 4). Protein carbonylation mainly results from the interaction of free radicals and amino acid side chains such as tyrosine, methionine, and lysine [32,33]. Therefore, the protein carbonyl content is also used to estimate protein oxidation [34]. In this study, the protein carbonyl content increased after air frying, and peaked at the end of air frying. Previous studies found similar results that protein carbonyl content in fish increased after boiling, steaming, microwaving, and roasting [35]. Lipids in fish are easily oxidized when exposed to the condition of oxygen and high temperature, and small molecules such as aldehydes, ketones, alcohols, and esters with aroma are produced. MDA as one of the main products of lipid peroxidation is often used as an indicator to evaluate the degree of lipid oxidation [36,37]. In this study, the MDA content significantly increased with the increase in air frying time. A similar result has been reported in *Arapaima gigas* after air frying and deep-fat frying [38].

### 2.4. Correlation Analysis among Chemical Properties and Key VCs during Air Frying

**Figure 5:**
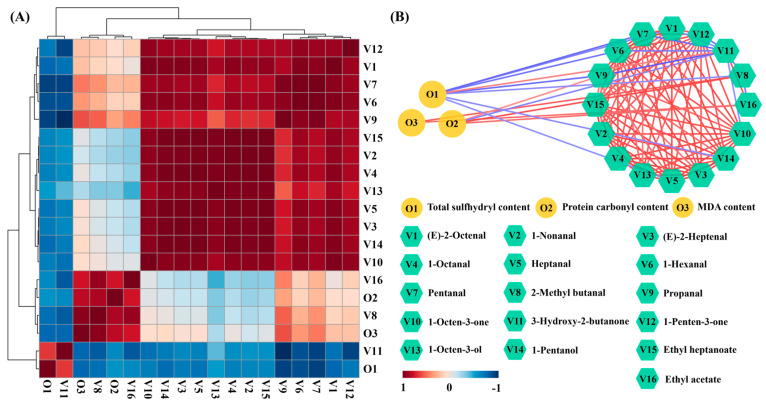
Correlation analysis among chemical properties and VCs during air frying of tilapia. (**A**) Correlation heatmap and (**B**) correlation network constructed by Pearson’s correlation coefficient (|r| > 0.6 and *p* < 0.05).

In order to analyze the change mechanism of key VCs, the correlation between the chemical properties and key VCs was preformed according to Pearson’s correlation test (Figure 5). As shown in Figure 5A, there were three evident clusters after the Pearson correlation analysis including VC-1 (V1, V2, V3, V4, V5, V6, V7, V9, V10, V12, V13, V14, and V15), VC-2 (V8 and V16), and VC-3 (V11). Most key VCs were clustered in the VC-1 and exhibited a positive relationship with each other, but exhibited a negative relationship with total sulfhydryl content. Moreover, the protein carbonyl content and MDA content exhibited a positive relationship with most key VCs. The correlation network was further drawn to clearly demonstrate the relationship between the chemical properties and key VCs (Figure 5B). The correlation coefficient |r| > 0.6 with *p* < 0.05 was considered to have a significant correlation between indicators. As shown in Figure 5B, the total sulfhydryl content was significantly negatively correlated with 2-methyl butanal (r = −0.623), 1-pentanol (r = −0.631), heptanal (r = −0.607), (E)-2-octenal (r = −0.662), 1-hexanal (r = −0.753), pentanal (r = −0.804), and propanal (r = −0.794), and the protein carbonyl content exhibited a significantly positive relationship with 2-methyl butanal (r = 0.869) and ethyl acetate (r = 0.785), manifesting the crucial role of protein oxidation in the formation of key VCs. Moreover, the MDA content exhibited a significantly positive relationship with 2-methyl butanal (r = 0.953), ethyl acetate (r = 0.789), and propanal (r = 0.624), showing that lipid oxidation made an important contribution to the formation of these pleasant VCs. Similar results have found that the carbonyl content and MDA content are significantly positively related with ethyl acetate and 2-methyl butanal in roasted tilapia [15]. These results indicated that the lipid and protein oxidation were closely related to the change in VCs during air frying of tilapia.

## 3. Materials and Methods

### 3.1. Air Frying of Tilapia

Live tilapia weighing about 1000 g was bought from a local supermarket in Guangzhou. The tilapia meat was soused with 0.6% complex phosphate (sodium tripolyphosphate/sodium hexametaphosphate = 1:1) and 6% salt for 40 min. Then, the soused meat was dried by hot air at 80 °C for 7 min and was then brushed with 2% soybean oil on the surface. After preheating at 190 °C for 10 min, the tilapia was roasted by air frying at 190 °C for 40 min in the air fryer oven (OD38AK813, Supor, China). The samples were taken after air frying for 0 min (CK), 20 min (A20), 30 min (A30), and 40 min (A40), respectively.

### 3.2. Determination of VCs by Quantitative HS-GC-IMS

The method of GC-IMS for identifying the VCs in tilapia referred to the previous study [24]. The mixture of 2.0 g ground tilapia and the internal standard substance (1 μg 4-methyl-2-pentanol) was put into a 20 mL headspace bottle and incubated at 60 °C for 15 min. The headspace gas (200 μL) was injected into the MXT-WAX column (30 m × 0.53 mm, 1.0 μm, RESTEK, Bellefonte, PA, USA) to analyze the VCs using the FlavourSpec^®^: Sensitive Analyzer (G.A.S., Dortmund, RPW, Germany). The GC condition was as follows: the initial flow velocity was maintained at 2 mL/min for 2 min, increased to 10 mL/min within 3 min, increased to 100 mL/min within 20 min, and then held at 100 mL/min for 5 min. Finally, the compounds were identified through the retention index and drift time. The VC concentration (C_x_) was calculated by the equation

(1) $$C_{x}=\frac{V_{x}\times W_{i}}{V_{i}\times W_{t}}$$

where C_x_ is the VC concentration (mg/kg), V_x_ is the VC peak volume, V_i_ is the 4-methyl-2-pentanol peak volume, W_i_ is the 4-methyl-2-pentanol weight (μg), and W_t_ is the tilapia weight (g).

The OAV of each VC was expressed as the ratio of the VC concentration to VC odor threshold. The VCs with OAV ≥ 1 were considered the key VCs in the air-fried tilapia.

### 3.3. Analysis of Oxidation of Proteins and Lipids

The protein carbonyl content assay kit and total sulfhydryl group content assay kit (Boxbio, Beijing, China) were used to test the total sulfhydryl and protein carbonyl content of air-fried tilapia according to their instructions, respectively. The assay method of MDA content referred to the previous study [39]. Briefly, 5.0 g tilapia was mixed with 50 mL trichloroacetic acid and shaken at 50 °C for 30 min. After filtration, thiobarbituric acid was added to the filtrate and kept at 90 °C for 30 min. The absorbance of the sample was measured at a 532 nm wavelength. The MDA content was calculated according to the standard curve.

### 3.4. Statistical Analysis

All experiments were repeated three times, and the data were expressed as the mean ± standard deviation. The difference in chemical properties of tilapia was analyzed by a one-way analysis of variance with the Tukey test. The principal component analysis (PCA) was used to analyze the similarity of VCs among different groups. The concentration heatmap of VCs and correlation heatmap among the key VCs and oxidative indexes of proteins and lipids were drawn using MetaboAnalyst 5.0. The correlation network was built using Cytoscape v.3.8.1 (https://cytoscape.org/, accessed on 15 August 2020) based on the Pearson’s correlation coefficient.

## 4. Conclusions

A total of 34 VCs were identified and quantified by quantitative GC-IMS, and 16 key VCs were determined according to OAV ≥ 1. Most VCs were improved by air frying and peaked in the A20 group. With the increase in air frying time, the total sulfhydryl content reduced, while the protein carbonyl and MDA content was enhanced, suggesting that the oxidation of the protein and lipid was promoted after air frying. The correlation network showed that the change in total sulfhydryl, protein carbonyl, and MDA was significantly correlated with most key VCs, especially 2-methyl butanal, ethyl acetate, and propanal, indicating that the oxidation of lipids and proteins after air frying made a contribution to the flavor improvement in tilapia. This study provides a useful reference for the volatile flavor improvement in a pre-cooked tilapia product.

## Figures and Tables

**Table 1:** Key VCs (OAV ≥ 1) during air frying of tilapia.

Volatile Compounds	OAV				Threshold (mg/kg)	Odor
	CK	A20	A30	A40		
(E)-2-Octenal	4.24	10.12	6.87	6.98	0.003	Fatty, Cucumber
1-Nonanal	73.77	232.29	126.49	98.26	0.001	Fatty, Floral, Lemon
(E)-2-Heptenal	0.99	3.93	2.04	1.80	0.013	Fatty, Fruity, Almond
1-Octanal	41.54	232.37	101.09	85.28	0.0007	Fruity, Fatty
Heptanal	21.52	119.59	56.98	50.16	0.003	Citrus, Green, Herbal
1-Hexanal	205.12	620.18	442.24	431.40	0.0045	Grassy, Fruity
Pentanal	13.88	44.24	33.50	33.46	0.02	Almond, Malty, Oil
2-Methyl butanal	74.52	117.42	180.35	231.05	0.001	Malty, Almond
Propanal	5.22	8.57	8.04	7.82	0.07	Floral
1-Octen-3-ol	27.65	78.67	34.10	32.02	0.0012	Mushroom
1-Pentanol	0.54	2.75	1.37	1.29	0.0015	Green
1-Octen-3-one	1725.71	4168.56	2965.51	2544.49	0.000005	Mushroom
3-Hydroxy-2-butanone	14.71	9.70	8.41	9.65	0.014	Buttery, Green
1-Penten-3-one	14.49	32.55	26.64	22.47	0.6	Green, Pungent
Ethyl heptanoate	0.25	2.04	0.80	0.58	0.17	Brandy, Fruity, Wine
Ethyl acetate	39.08	48.22	91.55	90.47	0.005	Fruity, Waxy, Floral

## Data Availability

The data presented in this study are included in the article.

## Supplementary Materials

The following supporting information can be downloaded at: https://www.mdpi.com/article/10.3390/molecules29184516/s1, Figure S1: Location of 56 VCs in topographic plot; Table S1: GC-IMS integration parameters of VCs during air frying of tilapia; Table S2: Change in VC concentration (mg/kg) during air frying of tilapia.
