# Supplementary material for: Identification of Key Volatile Compounds in Tilapia during Air Frying Process by Quantitative Gas Chromatography–Ion Mobility Spectrometry
Source: Molecules. 2024 Sep 23;29(18):4516. doi: 10.3390/molecules29184516 (PMC11434510; doi:10.3390/molecules29184516)
Supplement: Supplementary file 1 [file molecules-29-04516-s001.zip › molecules-3183840-supplementary.pdf]

Supplementary Data

Identification of key volatile compounds in tilapia during air frying process by quantitative GC-IMS

Tianyu Chen [1,2], Yong Xue [1], Chunsheng Li [2,*], Yongqiang Zhao [2], Hui Huang [2], Yang Feng [2], Huan Xiang [2], and Shengjun Chen [2,*]

[1] College of Food Science and Engineering, Ocean University of China, Qingdao 266003, China;

[2] Key Laboratory of Aquatic Product Processing, Ministry of Agriculture and Rural Affairs, National R&D Center for Aquatic Product Processing, South China Sea Fisheries Research Institute, Chinese Academy of Fishery Sciences, Guangzhou 510300, China;

* Corresponding author:

Chunsheng Li (E-mail: lichunsheng@scsfri.ac.cn)

Shengjun Chen (E-mail: chenshengjun@scsfri.ac.cn)

Fax: +86 20-82031851

Telephone: +86 20-89108310

* Correspondence: lichunsheng@scsfri.ac.cn (C.L.); chenshengjun@scsfri.ac.cn (C.S.);

Tel.: +86-186-7689-5850 (C.L.); +86-186-0207-3599 (C.S.)

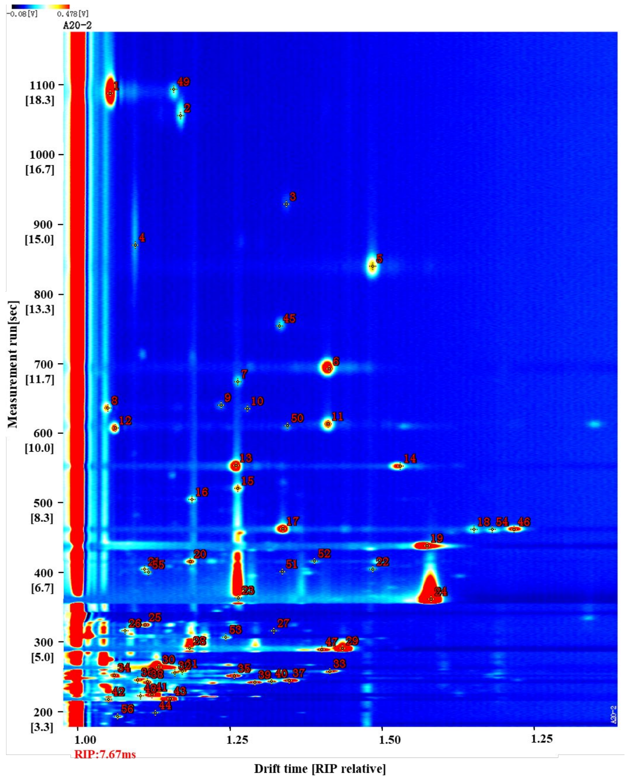

Figure S1. Location of 56 VCs in the topographic plot.

Table S1. GC-IMS integration parameters of VCs during air frying of tilapia.

No.	Compound	CAS	Molecule formula	MW	RI	RT (sec)	Dt (a.u)	Note
1	Acetic acid-M	C64197	C2H4O2	60.1	1489.8	1087.603	1.0562	Monomer
2	3-Octenol	C3391864	C8H16O	128.2	1479.9	1055.801	1.17182	
3	(E)-2-octenal	C2548870	C8H14O	126.2	1437.4	928.594	1.34526	
4	Furfural	C98011	C5H4O2	96.1	1415.8	870.012	1.09727	
5	1-nonanal	C124196	C9H18O	142.2	1403.8	839.047	1.48675	
6	Ethyl heptanoate	C106309	C9H18O2	158.2	1339.8	691.755	1.41524	
7	(E)-2-Heptenal	C18829555	C7H12O	112.2	1331.1	673.944	1.26526	
8	1-Hydroxy-2-propanone-M	C116096	C3H6O2	74.1	1311.7	635.508	1.05203	Monomer
9	1-Hydroxy-2-propanone-D	C116096	C3H6O2	74.1	1313.8	639.686	1.23762	Dimer
10	1-Octen-3-one	C4312996	C8H14O	126.2	1311.2	634.672	1.28105	
11	1-Octanal	C124130	C8H16O	128.2	1299.7	612.947	1.41333	
12	3-Hydroxy-2-butanone-M	C513860	C4H8O2	88.1	1296.5	607.098	1.06387	Monomer
13	1-Pentanol-M	C71410	C5H12O	88.1	1261.9	552.786	1.26229	Monomer
14	1-Pentanol-D	C71410	C5H12O	88.1	1261.3	551.951	1.53179	Dimer
15	2-Pentylfuran	C3777693	C9H14O	138.2	1239.4	520.314	1.26557	
16	(E)-2-Hexenal	C6728263	C6H10O	98.1	1227.8	504.406	1.19038	
17	Heptanal-M	C111717	C7H14O	114.2	1195.4	462.233	1.33984	Monomer
18	2-Heptanone	C110430	C7H14O	114.2	1194.8	461.493	1.65326	
19	1-Butanol-M	C71363	C4H10O	74.1	1153.6	414.881	1.18767	Monomer
20	(E)-2-Pentenal-M	C1576870	C5H8O	84.1	1143.4	404.152	1.11248	Monomer
21	ID_1	unidentified	*	0	1143.7	404.522	1.48659	
22	Hexanal-M	C66251	C6H12O	100.2	1101.8	363.089	1.26647	Monomer
23	Hexanal-D	C66251	C6H12O	100.2	1100.2	361.609	1.58261	Dimer
24	1-Propanol	C71238	C3H8O	60.1	1049.1	323.875	1.11339	
25	1-Penten-3-one-M	C1629589	C5H8O	84.1	1037.1	315.737	1.07987	Monomer
26	1-Penten-3-one-D	C1629589	C5H8O	84.1	1037.7	316.107	1.32445	Dimer
27	n-Pentanal-M	C110623	C5H10O	86.1	998.1	290.581	1.18676	Monomer
28	n-Pentanal-D	C110623	C5H10O	86.1	998.1	290.581	1.43677	Dimer
29	Ethanol	C64175	C2H6O	46.1	940.2	263.575	1.13603	
30	2-Methyl butanal-M	C96173	C5H10O	86.1	926.5	257.629	1.17386	Monomer
31	Isopropyl acetate	C108214	C5H10O2	102.1	921.7	255.572	1.16163	
32	2-Methyl butanal-D	C96173	C5H10O	86.1	926.2	257.501	1.41663	Dimer
33	2-Butanone-M	C78933	C4H8O	72.1	912.2	251.586	1.06195	Monomer
34	2-Butanone-D	C78933	C4H8O	72.1	911.3	251.2	1.26008	Dimer
35	Ethyl acetate-M	C141786	C4H8O2	88.1	896.1	244.9	1.10109	Monomer
36	Ethyl acetate-D	C141786	C4H8O2	88.1	895.1	244.515	1.34997	Dimer
37	Butanal-M	C123728	C4H8O	72.1	889.7	242.329	1.11699	Monomer
38	Butanal-D	C123728	C4H8O	72.1	887.5	241.429	1.29371	Dimer
39	ID_2	unidentified	*	0	892.6	243.486	1.32062	
40	2-Propanone	C67641	C3H6O	58.1	842	223.814	1.12432	

41	Propanal-M	C123386	C3H6O	58.1	824.9	217.514	1.05339	Monomer
42	Propanal-D	C123386	C3H6O	58.1	826.3	218.028	1.15429	Dimer
43	ID_3	unidentified	*	0	769.2	198.228	1.12983	
44	1-Hexanol	C111273	C6H14O	102.2	1368.3	753.797	1.33387	
45	Heptanal-D	C111717	C7H14O	114.2	1194.7	461.405	1.71854	Dimer
46	2-Pentanone	C107879	C5H10O	86.1	994.5	288.523	1.40351	
47	ID_4	unidentified	*	0	838.6	222.556	1.10541	
48	Acetic acid-D	C64197	C2H4O2	60.1	1491.7	1094.071	1.16014	Dimer
49	3-Hydroxy-2-butanone-D	C513860	C4H8O2	88.1	1298.3	610.349	1.34741	Dimer
50	Allyl sulfide-D	C592881	C6H10S	114.2	1139.8	400.408	1.33844	Dimer
51	1-Butanol-D	C71363	C4H10O	74.1	1154.5	415.937	1.39153	Dimer
52	2-Methyl-1-propyl acetate	C110190	C6H12O2	116.2	1023.4	306.65	1.24555	
53	ID_5	unidentified	*	0	1194.7	461.351	1.68349	
54	Allyl sulfide-M	C592881	C6H10S	114.2	1139.5	400.115	1.11865	Monomer
55	ID_6	unidentified	*	0	753.9	193.262	1.06805	
56*	4-Methyl-2-pentanol	C108112	C6H14O	102.2	1174.8	438.187	1.57626	Internal standard substance

Table S2. Change of VC concentration (mg/kg) and threshold value during air frying of tilapia.

No.	Volatile compound	CK	A20	A30	A40	Threshold value (mg/kg)
1	(E)-2-Octenal*	0.013±0.001^{c}	0.030±0.004^{a}	0.021±0.001^{b}	0.021±0.001^{b}	0.003
2	Furfural	0.199±0.021^{a}	0.183±0.024^{a}	0.185±0.015^{a}	0.191±0.017^{a}	3
3	1-Nonanal*	0.074±0.005^{c}	0.232±0.025^{a}	0.126±0.004^{b}	0.098±0.006^{ab}	0.001
4	(E)-2-Heptenal*	0.013±0.001^{c}	0.051±0.006^{a}	0.027±0.001^{b}	0.023±0.004^{b}	0.013
5	1-Octanal*	0.029±0.003^{c}	0.163±0.022^{a}	0.071±0.005^{b}	0.060±0.002^{b}	0.0007
6	(E)-2-Hexenal	0.047±0.010^{ab}	0.054±0.003^{a}	0.039±0.003^{b}	0.033±0.001^{b}	0.1
7	Heptanal*	0.065±0.007^{c}	0.359±0.029^{a}	0.171±0.018^{b}	0.150±0.012^{b}	0.003
8	1-Hexanal*	0.923±0.123^{c}	2.791±0.210^{a}	1.990±0.131^{b}	1.941±0.136^{b}	0.0045
9	Pentanal*	0.278±0.040^{c}	0.885±0.072^{a}	0.670±0.048^{b}	0.669±0.041^{b}	0.02
10	2-Methyl butanal*	0.075±0.011^{d}	0.117±0.001^{c}	0.180±0.014^{b}	0.231±0.003^{a}	0.001
11	Butanal	0.049±0.006^{c}	0.198±0.018^{a}	0.111±0.013^{b}	0.107±0.005^{b}	0.2
12	Propanal*	0.365±0.034^{b}	0.600±0.038^{a}	0.563±0.022^{a}	0.547±0.023^{a}	0.07
13	(E)-2-Pentenal	0.013±0.000^{c}	0.021±0.001^{ab}	0.024±0.000^{a}	0.017±0.003^{bc}	0.15
14	1-Hydroxy-2-propanone*	0.073±0.007^{c}	0.117±0.010^{b}	0.139±0.002^{ab}	0.159±0.016^{a}	80
15	1-Octen-3-one*	0.009±0.002^{c}	0.021±0.002^{a}	0.015±0.001^{b}	0.013±0.001^{b}	0.000005
16	3-Hydroxy-2-butanone	0.206±0.019^{a}	0.136±0.011^{b}	0.118±0.003^{b}	0.135±0.008^{b}	0.014
17	2-Heptanone	0.010±0.001^{a}	0.059±0.008^{b}	0.019±0.001^{b}	0.017±0.001^{b}	0.6
18	1-Penten-3-one*	0.017±0.003^{c}	0.039±0.001^{a}	0.032±0.004^{b}	0.027±0.001^{b}	0.0012
19	2-Butanone	0.139±0.011^{c}	0.275±0.009^{a}	0.214±0.012^{b}	0.235±0.011^{b}	50
20	2-Propanone	0.209±0.011^{c}	0.423±0.017^{a}	0.354±0.012^{b}	0.374±0.010^{b}	50
21	2-Pentanone	0.032±0.006^{c}	0.131±0.006^{a}	0.075±0.005^{b}	0.080±0.004^{b}	1.38
22	1-Octen-3-ol*	0.041±0.003^{b}	0.118±0.011^{a}	0.051±0.003^{b}	0.048±0.002^{b}	0.0015
23	1-Pentanol*	0.081±0.009^{c}	0.413±0.036^{a}	0.205±0.014^{b}	0.193±0.010^{b}	0.15
24	1-Butanol	0.095±0.010^{a}	0.086±0.011^{a}	0.110±0.006^{a}	0.086±0.012^{a}	5
25	1-Propanol	0.028±0.003^{c}	0.074±0.003^{a}	0.040±0.005^{b}	0.046±0.004^{b}	30
26	Ethanol	0.537±0.028^{b}	0.568±0.036^{b}	0.688±0.019^{a}	0.660±0.004^{a}	950
27	1-Hexanol	0.025±0.002^{b}	0.041±0.003^{a}	0.020±0.001^{c}	0.016±0.001^{c}	2.5
28	Ethyl heptanoate*	0.042±0.011^{c}	0.346±0.037^{a}	0.136±0.012^{b}	0.099±0.011^{b}	0.17
29	Isopropyl acetate	0.018±0.001^{d}	0.023±0.002^{c}	0.034±0.001^{b}	0.038±0.002^{a}	0.9
30	Ethyl acetate*	0.195±0.009^{c}	0.241±0.011^{b}	0.458±0.018^{a}	0.452±0.011^{a}	0.005
31	2-Methyl-1-propyl acetate	0.029±0.001^{a}	0.028±0.001^{ab}	0.027±0.001^{ab}	0.027±0.000^{b}	0.3
32	Acetic acid	0.956±0.152^{a}	0.816±0.037^{ab}	0.644±0.025^{bc}	0.569±0.025^{c}	99
33	2-Pentyl furan	0.014±0.003^{c}	0.105±0.009^{a}	0.048±0.003^{b}	0.045±0.005^{b}	0.27
34	Allyl sulfide	0.034±0.006^{b}	0.019±0.002^{b}	0.097±0.009^{a}	0.055±0.025^{b}	/

Data are shown as means ± standard. Values with different letters (a-d) on a row are significantly different based on Tukey's multiple range test ($p < 0.05$). The * means the key VCs with OAV ≥ 1 during air frying.
